# Physiopathology of the Brain Renin-Angiotensin System

**DOI:** 10.3390/life15081333

**Published:** 2025-08-21

**Authors:** Cristina Cueto-Ureña, María Jesús Ramírez-Expósito, María Pilar Carrera-González, José Manuel Martínez-Martos

**Affiliations:** Experimental and Clinical Physiopathology Research Group CTS-1039, Department of Health Sciences, School of Health Sciences, University of Jaén, E-23071 Jaén, Spain; ccueto@ujaen.es (C.C.-U.); mramirez@ujaen.es (M.J.R.-E.); pcarrera@ujaen.es (M.P.C.-G.)

**Keywords:** renin-angiotensin system, central nervous system, neurodegeneration, neuroinflammation, angiotensin II, angiotensin receptor, Alzheimer’s disease, Parkinson’s disease, cognitive impairment

## Abstract

The renin-angiotensin system (RAS) has evolved from being considered solely a peripheral endocrine system for cardiovascular control to being recognized as a complex molecular network with important functions in the central nervous system (CNS) and peripheral nervous system (PNS). Here we examine the organization, mechanisms of action, and clinical implications of cerebral RAS in physiological conditions and in various neurological pathologies. The cerebral RAS operates autonomously, synthesizing its main components locally due to restrictions imposed by the blood–brain barrier. The key elements of the system are (pro)renin; (pro)renin receptor (PRR); angiotensinogen; angiotensin-converting enzyme types 1 and 2 (ACE1 and ACE2); angiotensin I (AngI), angiotensin II (AngII), angiotensin III (AngIII), angiotensin IV (AngIV), angiotensin A (AngA), and angiotensin 1-7 (Ang(1-7)) peptides; RAS-regulating aminopeptidases; and AT1 (AT1R), AT2 (AT2R), AT4 (AT4R/IRAP), and Mas (MasR) receptors. More recently, alamandine and its MrgD receptor have been included. They are distributed in specific brain regions such as the hypothalamus, hippocampus, cerebral cortex, and brainstem. The system is organized into two opposing axes: the classical axis (renin/ACE1/AngII/AT1R) with vasoconstrictive, proinflammatory, and prooxidative effects, and the alternative axes AngII/AT2R, AngIV/AT4R/IRAP, ACE2/Ang(1-7)/MasR and alamandine/MrgD receptor, with vasodilatory, anti-inflammatory, and neuroprotective properties. This functional duality allows us to understand its role in neurological physiopathology. RAS dysregulation is implicated in multiple neurodegenerative diseases, including Alzheimer’s disease (AD), Parkinson’s disease (PD), and neuropsychiatric disorders such as depression and anxiety. In brain aging, an imbalance toward hyperactivation of the renin/ACE1/AngII/AT1R axis is observed, contributing to cognitive impairment and neuroinflammation. Epidemiological studies and clinical trials have shown that pharmacological modulation of the RAS using ACE inhibitors (ACEIs) and AT1R antagonists (ARA-II) not only controls blood pressure but also offers neuroprotective benefits, reducing the incidence of cognitive decline and dementia. These effects are attributed to direct mechanisms on the CNS, including reduction of oxidative stress, decreased neuroinflammation, and improved cerebral blood flow.

## 1. Introduction

The renin-angiotensin system (RAS) is a fundamental hormonal regulatory network that controls diverse physiological processes, including blood pressure, water and salt balance and cardiovascular homeostasis. Traditionally, it was considered a peripheral endocrine system, centered on the renin-angiotensin II (AngII)-AT1 receptor (AT1R) sequence, whose main role was to regulate circulating volume and systemic vascular resistance. However, research in recent decades has revealed the existence of a paracrine, autocrine and intracrine RAS, with several functional components in multiple tissues, including the brain and spinal cord [1,2].

The idea that the RAS has functions within the central nervous system (CNS) represents a model shift. All key elements of the system—enzymes, peptide precursors, and receptors—are present in specific brain regions, where they are involved not only in central cardiovascular regulation, but also in processes such as neurogenesis, synaptic plasticity, stress response, memory, inflammation, and neurodegeneration [3]. An altered brain RAS is intrinsically involved in the neuropathology of Parkinson’s disease (PD) and Alzheimer’s disease (AD), modulating oxidative stress, mitochondrial dysfunction and neuroinflammation [4,5]. In addition, brain RAS has been shown to be involved in the physiopathology of depression, anxiety and stress [6,7]. Therefore, interest in the RAS has increased not only because of its role in cardiovascular diseases such as hypertension or heart failure, but also because of its involvement in neurological and neurodegenerative disorders [6].

## 2. The Renin-Angiotensin System

The RAS comprises both systemic and tissue-specific axes with endocrine, paracrine, autocrine, and intracrine modes of action. In the classical systemic (endocrine) RAS, the first enzyme involved is the acid aspartate-protease renin [8], which cleaves circulating angiotensinogen to produce the decapeptide angiotensin I (AngI) [8]. Ang I is biologically nearly inactive and is converted by angiotensin-converting enzyme 1 (ACE1), a dipeptidyl carboxypeptidase highly expressed in pulmonary and endothelial tissues, into the potent vasopressor angiotensin II (AngII) [9]. Once formed, AngII serves as a substrate for various aminopeptidases—collectively termed angiotensinases—which modulate the spectrum and activity of downstream peptides mainly altering the ratios between their bioactive forms. Thus, aminopeptidase A (APA) removes the N-terminal aspartate of AngII to produce angiotensin III (AngIII), which retains vasopressor activity, while aminopeptidase N (APN) and aminopeptidase B (APB) convert AngIII into angiotensin IV (AngIV) by removing the N-terminal arginine [10,11]. AngII and AngIII exert their biological effects primarily via AT1R and AT2R [12,13], although AngIII appears to have a greater affinity for AT2R [14]. Other authors indicate that AngII and AngIII exhibit similar potency for both receptors [15]. Furthermore, it has been observed that AngIII, together with AngII and AT2R, are located in the inner membrane of mitochondria [15].

AngIII is considered a crucial peptide in the cerebral RAS for blood pressure control, with a tonic stimulatory effect on central blood pressure control, especially in hypertension models such as spontaneously hypertensive rats (SHR) and DOCA-salt rats [16,17,18]. The formation of AngIII is essential for the increase in blood pressure induced by AngII at the central level [17,19]. Studies with the APA inhibitor firibastat have shown that blocking the formation of AngIII in the brain leads to a reduction in blood pressure [20].

AngIII also plays a role in cardiovascular homeostasis through its sympathetic activity. It increases the release of vasopressin (arginine vasopressin) and activates sympathetic premotor neurons in the rostral ventrolateral medulla (RVLM). It also inhibits the baroreflex in the nucleus of the solitary tract (NTS) [16,18,21,22].

Ang III has also been associated with negative effects as it can exert a neurotoxic effect by activating AT1R, and it has proinflammatory properties [18,23,24]. Co-treatment with AngIII and the soluble form of aminopeptidase N (sANPEP) can synergistically increase the release of IL-1β in stimulated microglial cells [23]. It also induces the activation of STAT3 (signal transducer and activator of transcription 3), a protein that is essential in processes such as differentiation, proliferation, apoptosis, and inflammation in astrocytes [15]. It is important to note that AngIII concentration is increased in Alzheimer’s disease (AD) and is related to amyloid-beta (Aβ) and tau pathology [19,24,25].

Ang IV binds the AT4R, which has been molecularly identified as insulin-regulated aminopeptidase (IRAP). Intriguingly, AngIV has also been proposed to signal via the hepatocyte growth factor (HGF)/c-Met receptor axis [26], particularly in contexts of tissue repair and neoplastic growth. Furthermore, the HGF/c-Met pathway has been observed to attenuate neurodegenerative changes and prevent the loss of dopaminergic neurons [15,27]. Its functions also include processes such as improving learning and memory consolidation, cell proliferation and differentiation, and angiogenesis. Specifically, it facilitates N-methyl-D-aspartate (NMDA)-independent long-term potentiation (LTP) and promotes synaptogenesis in the hippocampus. Many of these functions overlap with those already known of the AngIV/AT4R/IRAP system [27,28,29]. The relationship between HGF/c-Met and AT4R/IRAP is a point of debate, as there are several functional similarities between the two systems.

At high concentrations, Ang IV can also bind to AT1R, and its binding to AT2R has even been reported [18]. The distribution of AT4R in the brain is remarkable, being found mainly in regions associated with cognitive, sensory, and motor functions, such as the hippocampus, cortex, basal ganglia, and basal nucleus of Meynert [27].

The AngIV/AT4R/IRAP axis is unique to the brain and promotes synaptic plasticity and glucose uptake, improving cognition, memory and learning. AngIV also stimulates nitric oxide (NO) release [18] and exerts a neuroprotective effect [6]. Ang IV analogs are capable of reversing spatial and temporal memory deficits. They have been observed to facilitate long-term potentiation (LTP) in the hippocampus, possibly through the activation of voltage-dependent calcium channels and the modulation of dopaminergic neurons. In murine models of Alzheimer’s disease, intracerebroventricular administration of AngIV promotes short-term memory and spatial learning [15,18,24,25].

In addition to its cognitive effects, AngIV is recognized for its neuroprotective properties [22]. It can also increase cerebral blood flow (CBF) without causing significant changes in systemic blood pressure [18]. It is an important modulator of glucose metabolism as it increases neuronal glucose uptake by modulating glucose transporter type 4 (GLUT4) trafficking [15,24,25]. It has been suggested that by inhibiting the catalytic activity of IRAP (its AT4R receptor), Ang IV prolongs the half-life of pro-cognitive peptides such as vasopressin, oxytocin, somatostatin, and endothelial nitric oxide synthase (eNOS). It has also been hypothesized that Ang IV modulates the release of serotonin, dopamine, and acetylcholine [18].

AngIV also exhibits anti-inflammatory and antioxidant effects [6]. It has been shown to suppress inflammation in the injured brain and reduce oxidative stress levels in the hippocampus [6,18]. However, its effects may be contextual; some studies have indicated that acute treatment with AngIV can activate the nuclear factor NF-κB and induce the expression of proinflammatory genes [30]. Furthermore, AngIV generated by sANPEP, when interacting with AT1R (which is predominantly upregulated in stimulated microglia), can promote proinflammatory activation of microglia and release of IL-1β [23]. In terms of neuronal plasticity, Ang IV increases synaptic efficiency in hippocampal neurons and promotes neurite growth and dendritic arborization [18].

Finally, AngIV is associated with cerebrovascular dysfunction and memory and recovery deficits, and it modulates ERK1/2 and PKC signaling cascades. It plays an important role in brain development and cognition processes [6,15,18,30].

The RAS also includes alternative biosynthetic pathways. For example, aspartyl aminopeptidase (ASAP) also participates in this metabolic cascade, converting AngI into the nonapeptide [des-Asp^1^]Ang I, which is subsequently converted by ACE1 into Ang III, thus bypassing AngII formation [31]. Another major axis involves ACE2, a carboxypeptidase homolog of ACE1 that degrades AngII to generate Ang(1-7), a heptapeptide with vasodilatory, anti-inflammatory, and antifibrotic properties [32] but also antioxidant and antithrombotic effects, in addition to neuroprotection [2,33]. The effects of Ang-(1–7) are mediated primarily through activation of MasR, a G protein–coupled receptor [34]. Notably, ASAP and APA can further metabolize Ang-(1–7) into Ang-(2–7), reducing its bioactivity. The ACE2/Ang(1–7)/MasR axis also counterbalances the classical ACE1/Ang II/AT1R axis. Finally, tissue and circulating carboxypeptidases, as well as non-specific proteases such as trypsin, chymotrypsin, and pepsin, contribute to the degradation of angiotensin peptides into inactive fragments, thereby fine-tuning RAS signaling dynamics [9,35].

In parallel, multiple local RAS operate independently of the systemic axis in a variety of tissues apart from the brain. These include the heart, vasculature, kidney, adrenal glands, adipose tissue, and even tumor microenvironments. In these compartments, all essential components of the RAS—angiotensinogen, renin (or renin-like enzymes), ACE1, and ACE2 and the several aminopeptidases—may be synthesized locally, allowing the autonomous generation and action of angiotensin peptides without the involvement of circulating renin. As an example, ACE2 plays a role in locally modulating nitric oxide and oxidative stress in blood pressure homeostasis and vascular injury [2,36].

This has led to the RAS being redefined as a set of opposing axes: the classical axis (ACE1/AngII-AT1R) for vasoconstrictor, proinflammatory and prooxidative actions, and the alternative axes (ACE2/Ang(1-7)-MasR and Ang II-AT2R) with vasodilator, anti-inflammatory and neuroprotective properties [37]. The ACE1/AngII-AT1R pathway has detrimental effects [4] including stimulating an oxidative environment and leading to inflammation, vasoconstriction and apoptosis [18,38], whereas the non-classical pathways ACE2/Ang(1-7)-MasR and Ang II/AT2R have beneficial effects against neurodegeneration, as seen in PD or AD [4], including reducing oxidative and inflammatory levels, promoting vasodilation and inactivating proliferative processes [18].

### 2.1. Alamandine: A Novel Component of the Renin-Angiotensin System

Alamandine is a heptapeptide discovered in 2013 [39] generated by the catalytic action of ACE2 on angiotensin A (AngA) or directly by the enzymatic decarboxylation of aspartic acid in Ang(1-7) [7,40,41,42]. Alamandine interacts selectively with its specific receptor, MrgD (Mas-related G-protein coupled receptor of the type D), a receptor different from MasR [40,41,42,43]. The MrgD receptor has been found in virtually all tissues/organs, including the brain [40] and, more specifically, in the cortex, hippocampus, amygdala, hypothalamus, habenular nuclei, striatum, pallidum, and the lateral and anterolateral regions of the periaqueductal gray (PAG), areas related to reward and the limbic system, suggesting a role in pain perception/modulation, synaptic plasticity, learning, memory, and cognition. [44,45]. It has also been identified in the vascular endothelium, arterial smooth muscle cells, and cardiomyocytes [42,45].

Alamandine and MrgD receptor form a counterregulatory axis within the RAS, similarly to the ACE2/Ang(1-7)/MasR axis. While the classic RAS axis (renin/ACE1/AngII/AT1R) exacerbates inflammation and fibrosis, the counterregulatory axes show anti-inflammatory and antifibrotic effects [42].

At the brain level, the impact of alamandine on mental health and cognitive functions is well known. Alamandine exerts an antidepressant effect. In transgenic rats with low levels of brain angiotensinogen (TGR), which exhibit depression-like behavior, intracerebroventricular (icv) infusion of alamandine significantly reduced immobility time in the forced swim test [40]. This effect was reversed by pretreatment with D-Pro7-Ang(1-7), but not with A779, confirming MrgD receptor mediation [40].

Alamandine also attenuates anxiety-like behavior. In TGR rats, dysfunction of the serotonergic system due to low levels of serotonin and its metabolites contributes to anxiety and depression behaviors [41]. Alamandite reversed the anxiety-like phenotype in the elevated plus maze test (increasing time and entries into the open arms) and in the novelty-suppressed feeding test, decreasing the latency to feed. These effects were dependent on MrgD receptor, not MasR [41].

Alamandine also improves spatial memory, as demonstrated in the Morris water maze test. Untreated depressed rats and those treated with A779 showed poor performance, while those treated with alamandine showed significant improvement [7].

Alamandine appears to reduce neuroinflammation and oxidative stress, which are pathological markers of depression. Significantly lower levels of proinflammatory cytokines (TNF-α, IL-1β, and IL-6) and oxidative stress (MDA) were observed in the hippocampus and prefrontal cortex of the alamandine-treated groups [7,46]. Alamandite increases BDNF levels and NMDA receptor expression, while decreasing GABA levels in the hippocampus and prefrontal cortex. These molecular changes correlated with improvements in cognitive abilities. BDNF levels are crucial for synaptic plasticity, learning, and memory, while elevated GABA levels are associated with cognitive dysfunction in depression [7]. Alamandine also reduces elevated serum cortisol and epinephrine levels, indicators of activation of the hypothalamic–pituitary–adrenal (HPA) axis and the sympathetic nervous system in rats with depression [7].

### 2.2. RAS Location in Central and Peripheral Nervous Systems

The brain RAS is a complex network that operates with remarkable independence from the circulatory system, although with crucial points of interaction, and performs its functions through its components in both vascular cells and non-vascular brain cells [27,47]. This means that angiotensins, their converting enzymes, and their receptors are present and active in both types of cells. Therefore, SRA in the vascular components of the brain, i.e., endothelial cells and vascular smooth muscle cells, modulates cerebral blood flow and BBB integrity [27,47]. Thus, ACE1 and ACE2 are predominantly found in the cerebral vascular endothelium, as well as in the choroid plexus [5,6,15,48,49,50]. APA is associated with the cerebral microvasculature, where AngII exerts direct effects through AT1R, expressed in endothelial cells and cerebral vascular smooth muscle cells [17]. Its activation is responsible for cerebral vasoconstriction and reduced cerebral blood flow [27,47,48]. As for AT2R, its presence in endothelial cells is still debated, and it generally counteracts the effects of AT1R by promoting vasodilation and vascular protection [14,47,48]. Similar function and location have been described for MasR and its ligand Ang(1-7) [15,47,48,50].

One of the reasons why the CNS RAS is anatomically and functionally distinct from the circulating system is that the blood–brain barrier (BBB) and the cerebrospinal fluid (CSF) barrier limit the diffusion of angiotensin peptides from the periphery into the brain. Although some studies suggested that the brain RAS derives from remnant levels of circulating AngII trapped in certain brain regions, which is why extremely low levels of (pro)renin and AngI appear in the brain, it is mainly thought that the BBB and CSF barrier restrict access to several RAS components so effectively, which underscores the need for their local synthesis [1,15,20,38,51,52]. Certainly, peripheral RAS may interact with brain RAS, especially in circumventricular organs lacking the BBB [15]. Therefore, RAS components are also present in brain cells, including neurons, astrocytes, oligodendrocytes, and microglia, and play a crucial role in both brain homeostasis and various neurological pathologies [19,53]. Thus, renin and angiotensinogen production take place in specific areas, such as the hypothalamus, pineal gland, cerebral cortex, and ependymal cells of the lateral ventricle [27,53]. Although the concentration of (pro)renin in the brain is low, it has been detected in neurons and astrocytes. PRR, on the other hand, is highly expressed in neurons and some microglial cells, allowing the activation of prorenin and the generation of AngII. In addition, it has been proposed that alternative enzymes, such as tonin, cathepsins B, D, and G, and chymase, can generate Ang II directly from angiotensinogen, without the need for AngI as an intermediary, particularly in the absence or limited release of renin.

Angiotensinogen is found in astrocytes at the nuclear level (approximately 90%) [27], and to a lesser extent in neurons and other glial cells. This precursor is secreted into the extracellular fluid of the brain and the CSF. Unlike renal production, central angiotensinogen production in the brain is not as influenced by blood pressure or circulating volume, suggesting a more autonomous autoregulatory mechanism in the CNS [3]. These components are found in both neurons and glial cells.

ACE1 is expressed in astrocytes and neurons, while ACE2 is found in neurons, astrocytes, and microglia. ACE1 is found in the area postrema, nucleus tractus solitarius (NTS), and hypothalamus, where it acts to convert AngI to the active form, Ang II [26,54]. ACE2 localizes to various brain areas, including the cerebral cortex and hippocampus [2,55]. These regions, along with the amygdala and prefrontal cortex, are particularly susceptible to chemical toxicities that can induce cognitive and behavioral abnormalities [55]. This complexity could be due to the conversion of Ang II to Ang(1-7) by ACE2 and the vasodilatory and anti-inflammatory properties of this peptide [37].

APA is located in both neuronal bodies and nerve fibers, where it is responsible for the formation of Ang III [17,42]. APA has been identified in numerous brain nuclei involved in the control of body fluid homeostasis and cardiovascular functions [49,51]. This includes areas such as the pituitary gland, the circumventricular organs, the median eminence, the arcuate nucleus, the choroid plexus, the paraventricular and supraoptic nuclei of the hypothalamus, the area postrema, the lateral reticular nucleus, and the nucleus of the solitary tract [47,50]. Similarly, APN has been identified in a small subset of oligodendrocytes, where it is responsible for forming Ang IV [17,42,47,50,51].

Angiotensin receptors (AT1R, AT2R, AT4/IRAP and MasR) are the main mediators of the physiological actions of the RAS in the CNS. The location and function of these receptors vary depending on the brain region and pathological conditions.

The AT1R is the main mediator of the classical effects of AngII, such as vasoconstriction, aldosterone release and sodium retention. This receptor is located in various brain regions, from those involved in cardiovascular regulation (solitary tract nucleus, paraventricular nucleus) to those related to cognition (cortex, hippocampus, basal ganglia) [5,15,47]. It has also been found in the mitochondria and nuclei of cells, forming part of an intracrine SRA [53]. AT1R appears in neurons, astrocytes, oligodendrocytes, and microglia [14]. The effects of AT1R activation include modulation of central blood pressure, regulation of social behavior, and involvement in mechanisms of neuroinflammation.

The AT2R is also located in neurons, astrocytes, oligodendrocytes, and microglia, although with predominantly neuronal expression [2,5,15]. Although less prevalent than AT1, it is involved in neuroprotective, anti-inflammatory, and vasodilatory processes. This receptor is localized in the brain in areas such as the hippocampus, cingulate cortex, thalamus, hypothalamus, brainstem and spinal cord, and its activation appears to counteract the adverse effects of the AT1R [56]. It has been suggested that AT2R may play an important role in neuroprotection under conditions of oxidative stress and in neurodegenerative diseases.

AT4R/IRAP is mainly found in neurons, although there are conflicting results regarding its presence in astrocytes. It is most abundant in the sensory and cognitive regions of the brain, such as the hippocampus, cortex, and basal ganglia [25,27,57].

The MasR, activated by Ang(1-7), has emerged as a key regulator in the neuroprotective effects of RAS. MasR is expressed on the membrane of endothelial cells, neurons, astrocytes, and microglia, and is widely distributed in the brain, especially in regions involved in cognition such as hippocampal dentate gyrus, piriform cortex and amygdala [18,58,59], and those involved in blood pressure control, such as the hypothalamus, cortex and brainstem. Regarding its presence in cardiovascular control centers, MasR appears in the nucleus tractus solitarius (NTS), rostral ventrolateral spinal cord (RVLM), caudal ventrolateral spinal cord (CVLM) and paraventricular nucleus (PVN) [18]. Its activation is associated with vasodilatory, neuroprotective and anti-inflammatory effects, suggesting that it may have a crucial role in the treatment of neurodegenerative diseases, such as AD and PD [26,37,60].

RAS is also present in the peripheral nervous system (PNS), where it plays a crucial role in the modulation of autonomic activity, inflammatory response, and control of vascular tone. The main components of the RAS in the PNS include the enzymes renin, angiotensinogen, and ACE, as well as the angiotensin receptors AT1R and AT2, located mainly on neurons and glial cells of the autonomic ganglia [53]. As in the CNS, the RAS in the PNS is activated in response to stimuli such as stress, hypoxia, and inflammation. Renin is produced locally in sympathetic and parasympathetic ganglia, and angiotensinogen is found in non-myelinating glial cells, participating in neurovegetative modulation and peripheral inflammatory processes [61], and in peripheral nerves, contributing to local activation of the RAS. This activation in the PNS is crucial for the modulation of cardiovascular response and sodium homeostasis [3]. AT1Rs are the most widely expressed receptors in the PNS, especially in autonomic ganglia, where their activation promotes noradrenaline release, which in turn affects cardiovascular function [53]. The AT2R in the PNS has a role in regulating vascular tone and modulating inflammation in peripheral tissues, although its involvement in neurovegetative responses is not completely clear.

However, the anatomical and cellular organization of the RAS in the CNS and PNS is complex and differs in several aspects because RAS is involved in functions as diverse as the regulation of cardiovascular homeostasis to the modulation of neurophysiological and behavioral processes. Therefore, the components of the RAS are distributed differently depending on the physiological needs of each region and how specific cellular interactions contribute to these functions [37].

### 2.3. Dysregulation of RAS Cascade and Pharmacological Intervention

It is widely recognized that dysregulation of the RAS cascade is implicated in cardiovascular diseases, hypertension, and diabetes [2,58,62,63]. Hypertension, in particular, is a well-established modifiable risk factor for dementia, contributing to cognitive decline through various mechanisms, including effects on cerebral perfusion, oxidative stress, inflammation and BBB integrity [14,15,27,48,57,64]. Both dementia and metabolic syndrome are indeed emerging global health threats [65]. While some studies suggest that the risk of incident dementia increases with each additional component of the metabolic syndrome, indicating a potential link [65], this relationship is complex and still uncertain due to conflicting findings in the literature. For instance, a meta-analysis of six longitudinal studies found no statistically significant pooled effect of metabolic syndrome on the risk of incident dementia [30,65]. This highlights that the association between metabolic syndrome and dementia remains to be fully elucidated, with some studies pointing to positive links, while others are potentially limited by sample size or follow-up duration [65].

In any case, the role of RAS in cerebral aging, AD, PD, and stroke has received increasing attention, given that pharmacological modulation of RAS with ACE inhibitors (ACEIs), AT1R antagonists (ARA-II), or MasR agonists could have benefits beyond blood pressure control [3,48]. Modulation of brain RAS by ARAs and ACEIs may be effective in reducing the risk of PD and its neuropathology, improving cognitive function and visuospatial memory in these patients [4]. As an example, the ARA-II telmisartan protects against cognitive decline and prevents stress-induced memory impairment [25]. Both telmisartan and perindopril, an ACEI, prevent impaired locomotor activity and anxiety behavior associated with mild traumatic brain injury [1,66,67,68,69]. Several epidemiological studies and clinical trials have also shown that patients treated with RAS modulators have a lower incidence of cognitive impairment and dementia compared to other antihypertensives [47]. The use of RAS inhibitors was also associated with lower tau tangle density, especially in people without diabetes [70]. Decreased Ang II activity has beneficial effects on the brain, including reduction of blood pressure and acetylcholine release [2]. ACE2 activators or enhancement of non-classical RAS pathways by ARAs and ACEIs improve 5HT synthesis and release in the brain, as well as neurogenesis [6,71]. AT2R and MasR activators can attenuate depression by increasing BDNF [6]. The alternative RAS pathway Ang(1-7)-MasR has beneficial effects, promoting vasodilation, anti-inflammatory and antioxidant actions, giving it potential therapeutic value for the treatment of brain trauma [47]. These effects could be due to direct mechanisms on the CNS, including reduced oxidative stress, decreased neuroglial inflammation, improved cerebral blood flow, and preservation of neuronal plasticity [72]. Drugs that cross the BBB and act on the RAS enhance the effects on cognition, confirming that they achieve the brain targets behind BBB [73]. In addition, ARAs, used chronically by millions of people, have shown a favorable safety profile.

Regarding the alandamine/MrgD receptor pathway, it represents a novel therapeutic target as a new antidepressant therapy [40,74], as well as a treatment for anxiety, neuropathic pain, and hypertension. However, further research is needed to identify the mechanisms and brain areas that mediate the antidepressant effect of the alamandine/MrgD receptor pathway [40], confirm the findings in additional models of anxiety, and include female rats in experimental protocols to address sex differences in depression and anxiety [41]. It is also necessary to fully understand how MrgD receptors influence pain processing, explore their therapeutic potential [44], and evaluate the pharmacokinetic properties and side effects of their administration, as few clinical studies have been conducted on this topic [42].

## 3. Mechanisms of Action of the RAS in the Nervous System

### 3.1. (Pro)Renin and PRR

Although the existence of active renin in the brain has been debated due to its low levels and the possibility that the renin detected corresponds to circulating renin trapped in the vessels [15,20,47,49,72], a specific isoform of the central nervous system called renin-b has been identified. This isoform, which lacks a signal peptide and is expressed mainly in neurons, appears to have its own functions in modulating the cerebral RAS [18,27]. In fact, studies in renin-b-deficient mouse models have shown sympathetic hyperactivity and alterations in baroreflex sensitivity, facilitating the onset of neurogenic hypertension, suggesting a potentially protective role [18]. Prorenin has been detected in the brain, and through the activation of PRR, to which both prorenin and renin bind, its enzymatic activity is enhanced by cleaving angiotensinogen and playing an essential role in the production of Ang II and local activation of the RAS. Relatively high levels of both prorenin and PRR have been found in the CNS [5,27,38,42,47,62].

PRR is widely expressed in neurons, some microglial cells, and key regions for cardiovascular control, such as the subfornical organ, the paraventricular nucleus, and the nucleus of the solitary tract, as well as in areas not directly related to cardiovascular function, such as the cerebral cortex and basal ganglia [15,27,42,47,62]. In addition to its catalytic role in AngII generation, it can initiate its own signaling pathways that induce pro-oxidative and pro-inflammatory effects independent of AngII. In any case, AngII, the main bioactive peptide in the system, acts primarily through AT1R, generating vasoconstriction, increased sympathetic tone, ROS production, and inflammation [27,42,62]. Overactivation of this axis promotes the development of neurogenic hypertension [4,6,18]. Cerebral AngIII also regulates central blood pressure, exerting tonic stimulatory control, especially demonstrated in hypertensive rat models [4,17,72]. The imbalance between the classic AngII/AT1R axis and the counter-regulatory axes, which promote vasodilation, antioxidant, and anti-inflammatory effects, contributes to hypertensive pathology [27]. The overexpression of PRR in the hypertensive human brain supports its role in this process. Furthermore, disruption of the BBB allows circulating AngII to penetrate normally protected brain regions, exacerbating RAS activity; activation of AT1R in cerebral endothelial cells may mediate this disruption [18,20,75].

In the context of stroke and cognitive disorders, chronic activation of the cerebral RAS and elevated AngII levels trigger neuroinflammation, oxidative stress, and age-related degenerative processes [4,72,73]. PRR overactivation contributes to cognitive impairment through the Ang II/AT1R axis [5,27]. In models of traumatic brain injury, increased (pro)renin mRNA expression has been detected, and pharmacological inhibition of renin with aliskiren has been shown to reduce oxidative stress, glial activation, and spatial memory deficits [47]. In addition, the PRR has been implicated in the progression of gliomas via the Wnt/β-catenin pathway [38]. Chronic dysfunction of the cerebral RAS has also been linked to neurodegenerative diseases such as Alzheimer’s and Parkinson’s, in which memory impairment and other cognitive functions are associated with alterations in mitochondrial morphology and an increase in ROS induced by sustained AngII activation via AT1R [2].

### 3.2. AT1R

AT1R is the main mediator of the “classical” and often deleterious effects of the RAS [4]. Activation of AT1R, which is coupled to G proteins (Gq/11), promotes protein kinase C (PKC) activation and intracellular calcium release. In addition, it stimulates the activation of NADPH oxidase (NOX), resulting in the generation of reactive oxygen species (ROS), exacerbating oxidative stress [18,22,58]. This pathway can also activate MAPKs (such as ERK1/2), and NRTKs and regulate the expression of transcription factors such as NF-κB [2]. AT1R activation can also enhance the activity of ADAM17, a metalloprotease that mediates the shedding or release of ACE2 from the cell surface, reducing its expression and protective function [55,75]. Ang II can activate NF-κB signaling through TLR4 expression, and the MD2/TLR4 complex is required for the proinflammatory effect of Ang II [4].

Specific activation of AT1R in the brain is associated with cerebral vasoconstriction, an increase in intracranial pressure, and a contribution to the autoregulation of cerebral blood flow under stress. At the neuronal level, AngII through AT1R may influence the release of neurotransmitters such as dopamine and glutamate [28]. Chronic overactivation of AT1R contributes to neuroinflammation through the production of ROS and proinflammatory cytokines (such as IL-1β and TNF-α) in neurons and glial cells (astrocytes, microglia) [14,18,23]. It is also associated with neurotoxicity, neuronal damage, BBB dysfunction and cell death [18,29]. This activation contributes to cognitive and memory impairment [2,64]. In the context of neurodegenerative diseases such as AD and PD, AT1R overexpression and excessive Ang II production exacerbate neuroinflammation and neurodegeneration [4,14]. AT1R activation can induce dopaminergic neuron death and alpha-synuclein aggregation [4,65]. In addition, Ang II can inhibit serotonin biosynthesis, a mechanism linked to depression [6].

### 3.3. AT2R

The AT2R generally counteracts the deleterious effects mediated by AT1R, although its expression in adults is more limited, increasing in pathological conditions [2].

AT2R induces the activation of protein phosphatases, such as SHP-1, PP2A and MKP-1, leading to reduced protein phosphorylation [15,49,55]. It also stimulates the nitric oxide (NO)/cyclic GMP (cGMP) system and phospholipase A2 (PLA2) activation [49]. Activation of AT2R has also been shown to increase brain-derived neurotrophic factor (BDNF) levels [15].

AT2R plays a crucial role in neuronal differentiation and regeneration, as well as in synaptic plasticity and brain development [14,15]. Its activation is associated with vasodilatory, anti-inflammatory and antioxidant effects, protecting against neuronal damage and improving cognitive function [2,18,29]. In brain injury models, AT2R stimulation improves spatial memory and attenuates cognitive dysfunction [14,15,25]. It can form functional heterodimers with AT1R, antagonizing its effects [49].

### 3.4. AT4R/IRAP

AT4R/IRAP is predominantly a mediator of memory and learning [15,18]. This axis is unique to the brain [18]. AngIV binds to AT4R/IRAP and inhibits its peptidase activity, which prevents degradation of neuropeptides involved in memory. Since AT4R/IRAP localizes to brain regions important for memory, such as the nucleus basalis of Meynert, hippocampus, and neocortex, its activation improves short-term memory, spatial learning, and cognition [18,19,24]. It promotes long-term synaptic plasticity (LTP) in the hippocampus. It has also been associated with modulation of dopaminergic neurons and the activation of voltage-dependent calcium channels. Also, it can stimulate nitric oxide (NO) release [15,18]. Furthermore, AngIV can reduce neuroinflammation in a dose-dependent manner in models of chronic cerebral hypoperfusion via the AT4R/IRAP, without altering blood pressure [18]. AT4R/IRAP also stimulates acetylcolysine release from the hippocampus and enhances glucose uptake [18,24].

### 3.5. MasR

MasR is the main Ang(1-7) receptor. Its effects are generally opposite to those of AT1R activation, and it is considered a key component of the RAS counter-regulatory axis [2,4]. MasR is coupled to several G proteins (Gq/G11, Gs/PKA/cAMP, Gαi/s, and Gα12), and its activation leads to the phosphoinositide-3-kinase (PI3K) pathway [22]. It promotes endothelial nitric oxide synthase (eNOS) activation and NO production [18,22,27]. In addition, it inhibits NF-κB activation and reduces MAPK signaling [2,21,22]. It also activates the phosphorylation of Akt.

MasR is widely distributed in the brain, especially in regions involved in cognition and blood pressure control, such as the hypothalamus, cortex and brainstem [18]. Its activation is associated with vasodilator, neuroprotective and anti-inflammatory effects [2]; it improves cerebrovascular function and cerebral blood flow, attenuates endothelial dysfunction, and inhibits proinflammatory polarization of microglia and their activation. MasR activation also plays a role in synaptic plasticity, learning and memory [18,27,49]. In this sense, MasR has been shown to be crucial for object recognition memory [27]. Ang(1-7) through MasR also enhances neurogenesis and serotonin biosynthesis [6,71].

### 3.6. MrgD Receptor

The MrgD receptor is a G protein-coupled receptor activated by alamandin, a peptide that can be derived from Ang(1-7) or AngA [42]. Its effects are generally similar to those of Ang(1-7), acting in opposition to AT1R [2,42]. MrgD receptor is coupled to G proteins (Gq/G11 and Gs), which involves activation of the PKA/cAMP pathway [22,42]. Activation of MrgD receptor by alamandin induces eNOS activation and subsequent NO production, which contributes to its vasodilatory effects [42]. It has also been suggested to attenuate PI3K/Akt and MAPK pathways [22]. Although it is less studied in the brain than other receptors, increased levels of MrgD receptor have been observed in the hippocampus and prefrontal cortex following treatment with alamandine [2]. High concentrations of the Mas-related receptor MrgE in the mitochondria of dopaminergic neurons indicate a protective mechanism against AT1R-induced oxidative stress [2,5]. Alamandin also shows anti-fibrosis, anti-inflammatory and anti-apoptotic effects, in addition to neuroprotection [42].

**Figure 1:**
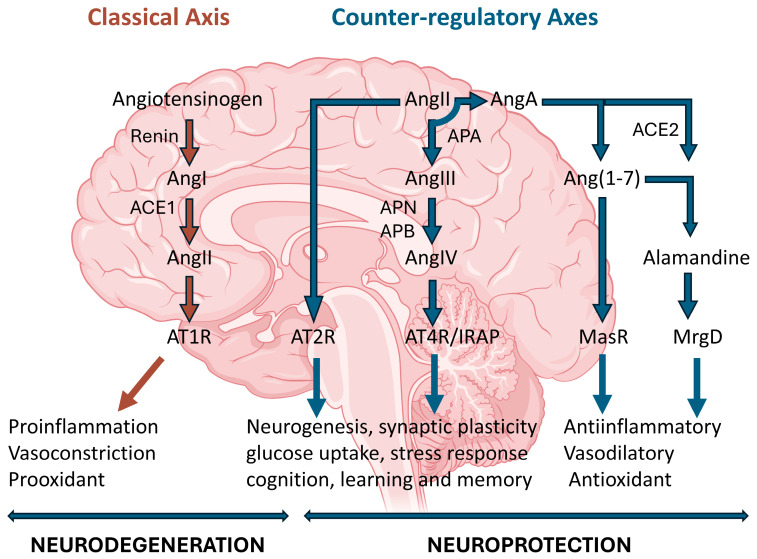
Classic and alternative pathways of the renin-angiotensin system. Renin converts angiotensinogen into angiotensin I (Ang I), which is transformed by angiotensin-converting enzyme 1 (ACE1) into angiotensin II (Ang II). Ang II acts mainly on the AT1 receptor, triggering vasoconstrictive, proinflammatory, and prooxidative responses associated with neuronal damage and cognitive impairment, and on the AT2 receptor (AT2R), with opposite effects. Alternative pathways include the formation of angiotensin IV (AngIV) by angiotensinases, angiotensin 1-7 (Ang(1-7)) by ACE2, and alamandine. AngIV acts on the AT4 receptor (AT4R/IRAP), Ang(1-7) binds to the Mas receptor (MasR), and alamandine binds to its MrgD receptor. These alternative pathways confer vasodilatory, anti-inflammatory, antioxidant, and neuroprotective effects. They also promote synaptic plasticity, memory, and neurogenesis. The dynamic balance between these pathways determines the net impact of the RAS on brain function, homeostasis, and neurological pathology.

The existence of these multiple mechanisms of action highlights the existence of a dynamic balance of the RAS in the brain and how it operates as a system of opposing axes. The classical axis (ACE/Ang II/AT1R) is associated with detrimental effects, such as vasoconstriction, inflammation and oxidative stress [4,18,29]. In contrast, counter-regulatory axes (Ang II/AT2R, ACE2/Ang(1-7)/MasR, Ang IV/AT4R/IRAP, and alamandin/MrgD receptor) exert beneficial and protective effects, promoting vasodilation, anti-inflammatory and antioxidant actions, neuroprotection, and improved cognition [2,4,18,24,29]. An imbalance toward AT1R axis overactivation contributes to various neurodegenerative and neuropsychiatric pathologies, whereas modulation of these axes (e.g., blocking AT1R or stimulating protective receptors) is considered a therapeutic strategy against different pathologies [14] (Figure 1).

## 4. RAS in the Aged Brain

As we age, the body’s physiological systems undergo a number of alterations, and the RAS is no exception. Brain aging is a complex process involving both structural and functional changes in the nervous system, including alterations in neurotransmitter and hormonal systems. With aging, changes in the expression and activity of RAS components are observed both centrally and peripherally, and these alterations can have significant implications for brain homeostasis, memory and cognition [2]. Dysregulation of the brain RAS, particularly increased AngII activity and decreased Ang(1-7), has been linked to cognitive and memory deficits observed in older adults [24,27,51]. Increased ACE1 expression has been found in memory-related regions such as the hippocampus and prefrontal cortex, resulting in elevated AngII levels that impair synaptic function and cognitive performance [26,54]. At the same time, blood AngII levels have been associated with reduced hippocampal and cortical volumes in cognitively normal elderly individuals [76].

Increased AT1R activity during aging is associated with enhanced vasoconstriction, neuronal inflammation, and impaired oxidative stress regulation [4,18,27]. Overactivation of the Ang II/AT1R axis leads to increased NADPH oxidase activity, superoxide production, and intracellular Ca^2+^ release, thereby promoting oxidative stress, neuroinflammation, and endothelial dysfunction through upregulation of NOX2 and ROS accumulation [3,47]. These processes contribute to cerebrovascular damage and chronic low-grade inflammation in the brain, driven by glial activation and the release of proinflammatory cytokines [18,27,51,53]. Age-related changes in the nigral RAS also lead to an increase in proinflammatory and pro-oxidative markers [3,27]. Moreover, AngII, through AT1R activation, contributes to cellular senescence by inducing telomere shortening and cell cycle arrest—effects that can be reversed by certain pharmacological agents [27].

The chronic activation of RAS also contributes to neuronal damage, synaptic dysfunction and impaired plasticity, all of which negatively affect learning and memory [37]. In particular, excessive AT1R activation by Ang II disrupts synaptic plasticity in key brain areas such as the hippocampus and prefrontal cortex [37]. AngII has been shown to potentiate NMDA receptor currents in a subpopulation of pyramidal neurons, and this effect can be reversed by AT1R—but not AT2R—antagonists, indicating a specific role of AT1R and its interaction with dopaminergic signaling [28]. Furthermore, AT1R blockade enhances dopamine reuptake kinetics, potentially contributing to improved cognitive performance [77]. In parallel, alterations in BDNF expression and increased ROS production further contribute to cognitive impairment during aging [2]. It has also been observed that continued RAS activation, particularly in the context of cardiovascular comorbidities, promotes apoptotic death of hippocampal neural stem cells via mitochondrial ROS generation, leading to memory deficits [2].

Animal models with altered ACE2 expression present additional evidence linking RAS dysfunction to cognitive decline. They show impaired neurogenesis and deficits in serotonin synthesis [2]. Genetic variants in RAS components are associated with accelerated hippocampal and cortical atrophy and with cognitive deterioration in the elderly [6,76]. Cognitive impairment has also emerged as a relevant concern in COVID-19 survivors, suggesting a broader role of RAS imbalance in post-viral and age-associated neurological decline [2].

In contrast to the deleterious effects of AngII, Ang(1-7), acting via the MasR, plays a neuroprotective role, but this axis is also compromised during aging. A significant reduction in Ang(1-7) levels has been observed in aging brains, which correlates with an increase in proinflammatory markers and the loss of the anti-inflammatory and antioxidant properties of this peptide [51]. Ang(1-7) has been shown to reduce microglial activation and inflammatory cytokine release, while preserving synaptic function and promoting neuronal plasticity in the hippocampus [3]. It also enhances recovery following traumatic brain injury in murine models [51,68]. Moreover, the aging process is associated with a decline in AT2R expression [30] and decreased ACE2 activity [37], further exacerbating the imbalance between AngII and Ang(1-7) and promoting neuroinflammation, neuronal apoptosis, and cognitive dysfunction [18,38,51]. The loss of compensatory mechanisms mediated by Ang(1-7) and AT2R may therefore contribute to age-related neurodegenerative processes. Synaptic plasticity, essential for learning and memory, becomes increasingly vulnerable to the chronic inflammatory and oxidative environment created by an overactive RAS. The dysfunction of this system extends to its interactions with ion channels such as calcium channels, which are critical for long-term potentiation (LTP), one of the cellular foundations of memory formation [18,38,51].

## 5. RAS and Neuroinflammation: Implications in Neurodegeneration

Neuroinflammation is a key process in the pathogenesis of various neurodegenerative diseases and is characterized by the chronic activation of glial cells and the release of proinflammatory mediators in the brain. This phenomenon is closely associated with the progression of disorders such as AD, PD, multiple sclerosis, and amyotrophic lateral sclerosis (ALS) [37,78]. In AD, AngII-mediated neuroinflammation contributes to β-amyloid plaque accumulation and neuronal dysfunction, while in PD, microglial inflammation exacerbates dopaminergic neuronal damage and disease progression [20,37]. RAS plays a crucial role in the regulation of neuroinflammation, and its chronic activation promotes a pro-oxidative and pro-inflammatory environment that increases neuronal vulnerability and accelerates neurodegeneration [2,4].

Under pathological conditions, increased AngII levels in the brain activate microglia—the resident immune cells of the CNS—leading to the release of proinflammatory cytokines such as interleukin-1 beta (IL-1β), interleukin-6 (IL-6), and tumor necrosis factor-alpha (TNF-α), which further amplify neuroinflammation and neuronal injury [2,15,37,50]. AngII also stimulates the production of cyclooxygenase-2 (COX-2), contributing to sustained inflammatory responses [2]. Chronic high-grade inflammation driven by AngII occurs mainly via AT1R activation, which triggers key inflammatory pathways including the transcription factor NF-κB [50]. Activation of NF-κB enhances the release of proinflammatory mediators and oxidative stress, further promoting neuronal dysfunction and disease progression [2,50].

Glial cell activation, particularly that of microglia and astrocytes, is a central feature of RAS-mediated neuroinflammation [2,14,18,23]. AngII induces microglial polarization toward the proinflammatory M1 phenotype, characterized by increased production of cytokines and reactive oxygen species [2,4,50]. Moreover, there is significant intercommunication between microglia and astrocytes through the brain RAS, contributing to the amplification of the inflammatory response [2,4,76]. AngII can also compromise the integrity of the BBB, facilitating the infiltration of peripheral immune cells and exacerbating neuroinflammation. In murine models, chronic subcutaneous infusion of AngII has been shown to increase BBB permeability, activate microglia, induce myelin loss, and impair memory performance [2,18].

In contrast to the proinflammatory effects of the classical ACE1/AngII/AT1R axis, the counter-regulatory axes ACE2/Ang(1-7)/MasR and the AT2R exert anti-inflammatory and neuroprotective actions [2,4,15,18,47,51]. These receptors mitigate oxidative stress and neuroinflammation by suppressing NADPH oxidase activity and inhibiting key inflammatory pathways such as NF-κB [2,50]. Activation of AT2R and MasR has been shown to reduce TNF-α levels and increase the release of the anti-inflammatory cytokine IL-10 in both astrocytes and microglia [57]. Furthermore, Ang(1-7) and AT2R promote microglial polarization from the M1 to the neuroprotective M2 phenotype, contributing to the resolution of inflammation and restoration of neural homeostasis. AT2R and MasR can also form heterodimers that inactivate AT1R and enhance CX3CR1 expression, further contributing to the inhibition of microglial activation [2,4]. MasR activation by Ang(1-7) also induces phosphorylation of protein kinase B (AKT), which suppresses NF-κB signaling and leads to a decrease in proinflammatory cytokine production. In addition, Ang(1-7) and AngIV stimulate the release of nitric oxide (NO), exerting antioxidant and neuroprotective effects [2]. AT4R has also been implicated in anti-inflammatory responses, as well as in synaptic plasticity and glucose uptake. Mice deficient in AT4R display severe deficits in spatial reference and object recognition, underscoring its relevance in cognitive function [18,24].

The modulation of the RAS toward a favorable balance between AngII and Ang(1-7) is increasingly considered a promising therapeutic strategy to reduce neuroinflammation and mitigate neuronal damage in neurodegenerative diseases [3]. Pharmacological interventions targeting this system, including AT1R antagonists and MasR agonists, have shown potential to reduce microglial activation, restore brain homeostasis, and slow the progression of diseases such as AD and PD. As stated below, accumulating evidence suggests that RAS dysregulation may also play a role in psychiatric disorders such as depression, reinforcing the broader implication of this system in CNS physiopathology [6,7,16].

## 6. RAS and Neuropsychiatric Disorders: Depression, Anxiety and Stress

Neuropsychiatric disorders, such as depression, anxiety and post-traumatic stress disorder (PTSD), are common pathologies that affect a large part of the population. Although the underlying mechanisms of these disorders are complex, the RAS has been identified as playing a relevant role in the modulation of emotions and behavior [6]. RAS dysfunction, especially overactivation of AngII and lack of the protective effects of Ang(1-7), is involved in the physiopathology of these disorders [2,4]. The underlying mechanisms include oxidative stress, mitochondrial dysfunction and neuroinflammation. Taken together, these processes can induce apoptotic death of hippocampal neural stem cells [2,4,6,18,22,48,55]. It should be remembered that the brain is particularly vulnerable to oxidative stress due to its high metabolic activity and low antioxidant capacity [2]. Additionally, RAS dysregulation also reduces serotonin and BDNF levels [2,6], with direct effects on modulating neuronal activity in key brain areas for emotional regulation, such as the limbic system, prefrontal cortex, hypothalamus, hippocampus and amygdala [6,18,22]. In fact, AngII modulates the activity of neurotransmitter systems involved in emotional regulation through serotonergic and dopaminergic systems, altering their balance and contributing to mood disorders [6]. In experimental studies, it has been shown that overstimulation of AT1Rs by AngII can induce anxious and depressive behavior in animal models [6]. In humans, elevated levels of Ang II have been observed to be associated with an increased risk of developing depression and anxiety [26,54].

Unlike AngII, Ang(1-7) exerts anxiolytic and antidepressant effects. This peptide has the ability to regulate the activity of the amygdala and hypothalamus, reducing the release of stress hormones such as cortisol and adrenaline [2,4]. MasR activation by Ang(1-7) has beneficial effects in reducing anxiety and depression and has been shown to improve emotional response in animal models of these disorders [3]. The administration of MasR agonists could be an effective strategy to treat neuropsychiatric disorders, improving emotional well-being and reducing symptoms associated with depression, anxiety and stress [37].

## 7. RAS in Neuropathic Pain: Therapeutic Implications

Neuropathic pain is a pathological condition characterized by chronic pain resulting from injury or dysfunction in the nervous system. This condition is often associated with alterations in pain modulation in the brain and spinal cord. The RAS has a relevant role in pain modulation, and its dysfunction could contribute to the perpetuation of neuropathic pain [50]. AngII is a key modulator in central sensitization, a pathological process in which the nervous system becomes more sensitive to pain. Activation of AT1Rs by Ang II in the central nervous system (CNS) increases the excitability of sensory neurons and promotes the release of inflammatory mediators that sensitize the pain pathways. This process contributes to the exacerbation of neuropathic pain, causing affected individuals to experience pain more intensely and prolonged [26,54].

In contrast, MasR activation by Ang(1-7) has analgesic effects, inhibiting central sensitization and reducing neuronal excitability in brain areas related to pain perception. Ang(1-7) has been shown to reduce the release of excitatory neurotransmitters, such as glutamate, and promote the release of neuroprotective factors that help restore balance in pain pathways [37]. Similarly, AT2R antagonists have emerged as potential novel analgesics. Preclinical investigations have evaluated the efficacy of EMA 300 and EMA 200 in animal models of neuropathic pain [50] and in the unilateral chronic constriction injury model of the sciatic nerve. These compounds have been shown to inhibit P38 and p42/p44 MAPK kinases, which are associated with decreased bradykinin and substance P metabolism [50]. The EMA 401 molecule has also been investigated in patients with postherpetic neuralgia, a condition characterized by chronic neuropathic pain, underscoring the brain RAS as a putative target for the development of new pharmacological strategies in the management of neuropathic pain [15,25,37,50].

## 8. RAS-Targeted Therapies in Neurological Diseases

As stated before, cerebral RAS modulation presents significant therapeutic potential for the treatment of various neurodegenerative and neurological diseases, psychiatric disorders, and neuropathic pain. As we better understand the effects of the different components of the RAS in the brain, new opportunities are emerging to develop more targeted and effective therapies.

Currently, there are several pharmacological treatments that modulate the RAS, such as ACEIs, ARAs and direct renin inhibitors. These drugs are commonly used to treat hypertension and cardiovascular disease, but also show potential for the treatment of neurological disorders. Thus, ACEIs and ARAs have been shown to be effective in reducing the risk and neuropathology of PD and also have neuroprotective effects and improve cognitive function and visuospatial memory in these patients [4]. These compounds may also decrease the progression of neuroinflammation by inhibiting proinflammatory cytokines [4]. The AT1R antagonist candesartan reduces TGF-β1 expression in traumatic brain injury [66,67,68,69,72] and has neuroprotective effects [47]. Telmisartan modulates glial activation [15,25]. More recently, irbesartan has demonstrated significant improvements in memory performance, which correlates with an increase in dendritic spine density in the hippocampus, indicating an improvement in synaptic plasticity. Furthermore, it activates cell survival pathways by increasing Creb1 and BDNF expression and is positioned as a promising multitarget therapeutic strategy for AD by modulating synaptic and mitochondrial dysfunction, oxidative stress, apoptosis and neuroinflammation [5,25]. Irbesartan has also been observed to positively influence amyloid precursor protein (APP) processing enzymes, increasing ADAM-10 and decreasing BACE-1 [25]. It has also demonstrated potent anti-inflammatory and antioxidant effects, reducing biomarkers of oxidative stress [6], decreasing proinflammatory cytokines such as Il1β and Il6, and enhancing microgliosis and astrogliosis by reducing the activation of these glial cells. Another important effect is the restoration of BBB integrity, decreasing leakage markers and increasing claudin-5 [25]. Additionally, irbesartan restores mitochondrial bioenergetics by increasing levels of oxidative phosphorylation complexes (OXPHOS) and improving insulin resistance, which could be related to its function as a selective modulator of peroxisome proliferator-activated receptor gamma (PPARγ) [15,25,47].

Other ACEIs and ARBs increase brain 5HT and improve depressive symptoms [6], whereas RAS inhibitors were associated with a measure of insulin signaling, specifically with a lower level of AKT1 phosphorylation in the brain [70]. Firibastat, a central APA inhibitor prodrug, prevents the conversion of AngII to AngIII in the brain and upregulates brain ACE2, which could be particularly useful in African American and obese patients [16]. Orally administered firibastat has been shown to cross intestinal, hepatic and blood–brain barriers [17]. Antisense oligonucleotides or small interfering ribonucleic acids (siRNAs) that suppress hepatic angiotensinogen for weeks to months are also being developed, which could overcome treatment adherence problems [16]. Multistage delivery vectors (MDVs) and nanoparticles are being developed for RAS inhibitor therapy to deliver drugs directly into the brain using blood–brain barrier transporters and cell-specific targeting [19].

However, more research is still needed to fully understand the effects of these treatments on the brain and their efficacy in various neurological conditions. The fundamental neuroprotective mechanisms of ACEIs and ARAs have not been fully elucidated, and preclinical and clinical trials are needed to verify these claims [4,37].

One of the main challenges is the need to develop treatments that can precisely and selectively modulate the AngII and Ang(1-7) pathways without causing unwanted side effects. For example, although AT1R inhibition by ARAs reduces AD-associated pathology, it increases the likelihood of lowering blood pressure [14]. Although telmisartan may mitigate behavioral alterations, it may also impair spatial working memory [79]. Furthermore, understanding of the long-term effects of RAS modulation in the brain is still limited, and more research is needed to ensure the safety and efficacy of these therapeutic approaches, also taking into account that the nanomolar to micromolar concentrations of angiotensins applied in experiments might be supraphysiological [28].

## 9. Conclusions

The RAS represents much more than a peripheral cardiovascular control mechanism, constituting a complex neurohormonal system with profound implications for brain function and neurological pathology. The evidence presented demonstrates that the brain RAS operates as an independent system with capacity for local synthesis of its components, which challenges the traditional conception of its exclusive dependence on the peripheral circulation. The identification of Ang II-AT1R versus Ang(1-7)-Mas/Ang II-AT2R antagonistic axes provides a fundamental conceptual framework for understanding how imbalance between these pathways contributes to the physiopathology of neurological/neurodegenerative diseases. The epidemiological and clinical findings demonstrating the neuroprotective benefits of ACEIs and ARA-II beyond their antihypertensive effects represent a significant advance in the field of preventive neurology. The observed reduction in the incidence of cognitive impairment and dementia in patients treated with these drugs suggests that RAS modulation could be a viable preventive therapeutic strategy for neurodegenerative diseases. Understanding the dual mechanisms of the RAS opens new therapeutic avenues. The development of specific MasR agonists and selective modulators of the Ang(1-7) pathway could allow for more precise and effective interventions. The identification of new compounds and the development of brain-targeted delivery systems represent promising advances in this field. Despite advances, significant challenges remain that require additional research. The fundamental neuroprotective mechanisms of ACEIs and ARBs are not fully elucidated. The need to develop treatments that precisely and selectively modulate RAS pathways without unwanted side effects represents a major challenge. In addition, understanding the long-term effects of RAS modulation in the brain requires more extensive longitudinal studies. Rigorous preclinical and clinical trials are needed to fully validate claims about the therapeutic potential of RAS in neurological diseases. Future studies should focus on (1) fully elucidating the molecular mechanisms of neuroprotection; (2) developing specific biomarkers to monitor brain RAS activity; (3) designing controlled clinical trials that evaluate the efficacy of RAS modulation in specific neurological patient populations; and (4) exploring RAS interactions with other neurotransmitter and neurohormonal systems. The presented results suggest that the selection of antihypertensives in patients at risk for cognitive impairment should consider not only cardiovascular effects, but also neuroprotective potential. This could influence future clinical guidelines for the management of hypertension in populations at neurological risk. As a whole, the RAS emerges as a crucial integrative system at the interface between cardiovascular and neurological health, offering new therapeutic opportunities to address the growing problem of neurodegenerative diseases in an aging population. Translation of these findings into clinical practice requires a multidisciplinary approach integrating neurology, cardiology, and pharmacology to optimize the therapeutic benefits of the RAS on brain health.
